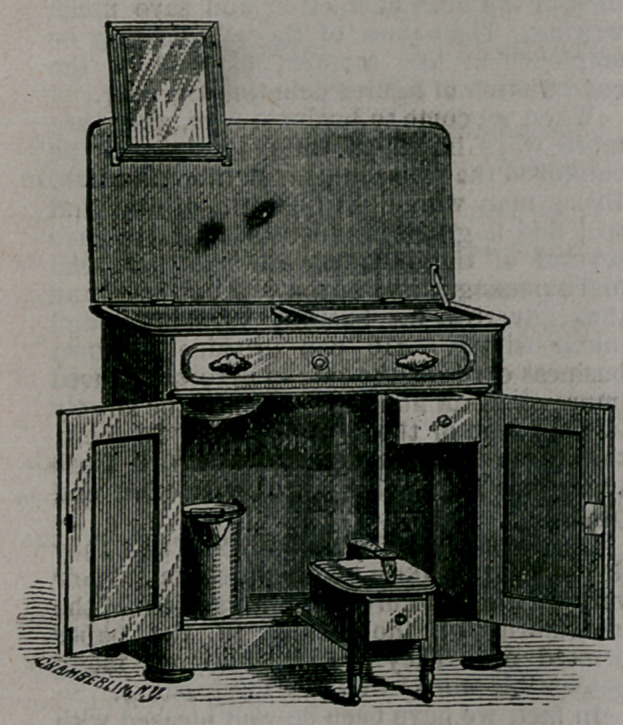# A Genuine Convenience

**Published:** 1875-10

**Authors:** 


### A GENUINE CONVENIENCE.

In these days of light housekeeping, when the utmost effort is made to hide all unsightly objects in the sleeping apartment, so that the bedroom may be used as a sitting-room as well, inventive genius is taxed to the utmost to disguise the leading articles of chamber use, and to so transform them that their presence by day in the living apartment shall not be deemed objectionable. Thus we have sofas which are transformed into comfortable beds, bedsteads which may be turned up against the wall to appear like elegant wardrobes, and a host of other contrivances, by the aid of which the chamber quickly becomes an attractive boudoir.

One of the most efficient and ingenious devices for the purpose indicated, may be seen at the Fair, and should be carefully examined by all. It stands on the right as you enter, near the man who exhibits a valuable self-lighting gas-cock. We allude to a patent washstand, the invention of Schwarz Brothers, whose place of business is at 191 and 193 Stanton Street, New York. It will well repay an examination on the part of all good housekeepers.

It is a very neat piece of furniture, as our cut indicates. The specimen on exhibition has a marble top, which is easily thrown back by the aid of a spring concealed. When lifted, it reveals a fixed bowl with a neat silver faucet conveniently situated. A tank holding and hiding abundance of water, is arranged at the right of the bowl, and a spout, flush with the side of the bowl, conveys the water from the tank. A plug is provided for the hole in the bottom of the bowl, with chain attached, precisely as the plumber adjusts these things where a running water-supply exists. The waste water escapes into a pail under the bowl. A mirror, which shuts down with a spring, is attached to the under side of the top. Underneath, a a portion of the space is occupied by a neat boot-blacking stand, which can be thrown outward for use when required, and which contains a drawer for the blacking utensils.

On the whole, no object in the Fair pleases us more than this. In country houses and in apartments not supplied with running water, it is exactly the thing. No bowl and pitcher disfigure the apartment. Only a neat, panelled, black-walnut, marble-topped closet or commode is visible; and this may be adorned with statuettes or ornaments, which can be removed when the fixed bowl is needed for use. The top, when thrown back, protects the wall from the drops which are sprinkled about in the various processes of ablution. No crockery or china is visible, and therefore there is none to be lifted and cared for. In effect, the room furnished with this elegant device is supplied with running water.

The price of the sample at the Fair is $30. Other styles are made, down to $8. We feel sure that our friends will thank us for calling their attention to an article of household furniture, which, when examined, will manifest itself as almost a necessity in thousands of localities which are unsupplied with running water.

## Figures and Tables

**Figure f1:**